# Using Concepts of Photovoice to Engage in Discussions Related to Burnout and Wellbeing

**DOI:** 10.3390/ijerph21020192

**Published:** 2024-02-07

**Authors:** Gretchen Macy, Whitney Harper, April Murphy, Kim Link, Austin Griffiths, Shwe Win, Ashley East

**Affiliations:** 1 Center for Environmental and Workplace Health, Department of Public Health, Western Kentucky University, 1906 College Heights Blvd, Bowling Green, KY 42104, USA; shwe.win437@topper.wku.edu (S.W.); ashley.east607@topper.wku.edu (A.E.); 2 Department of Social Work, Western Kentucky University, 1906 College Heights Blvd, Bowling Green, KY 42104, USA; whitney.harper@wku.edu (W.H.); april.murphy@wku.edu (A.M.); austin.griffiths@wku.edu (A.G.); 3 Lifeskills Center for Child Welfare Education and Research, Western Kentucky University, 1906 College Heights Blvd, Bowling Green, KY 42104, USA; 4 Institute for Rural Health, School of Nursing and Allied Health, Western Kentucky University, 1906 College Heights Blvd, Bowling Green, KY 42104, USA; kim.link@wku.edu

**Keywords:** workplace stress, burnout, well-being, photovoice

## Abstract

The purpose of this study was to identify essential skills and abilities for mitigating job-related stressors and preventing burnout while also establishing connections between students and community health workers to provide students with a deeper comprehension of the challenges inherent to their future professions. Ten community health workers were interviewed and asked to present photographs that explored sources of burnout and promotions of well-being. The photographs along with quotes were displayed in a gallery style exhibit for students to view and talk with the community health workers and complete a survey. Using thematic analysis, the interviews resulted in four common factors that contribute to burnout: (1) workload demands, (2) unrealistic exceptions, (3) amount of time dedicated to care, and (4) lack of work–life balance. The themes that emerged from student responses were (1) learning self-care practices, (2) gaining insight into the need for self-care, (3) a sense of connection, and (4) exposure to different healthcare careers. This study demonstrates the importance of connecting students with community health workers. It increases understanding of the demands of their future professions as well as resources and engagement opportunities available to them as a part of their respective professional community.

## 1. Introduction

Mayo Clinic defines burnout as, “a state of physical or emotional exhaustion that also involves a sense of reduced accomplishment and loss of personal identity” [1]. Studies have shown that health service professions are some of the most vulnerable to occupational burnout. Consistent exposure to physical and mental suffering and death is a major contributor to burnout among these professions [2]. The COVID-19 pandemic further increased the likelihood of burnout among health services workers leading to increased job demands, limited individual autonomy at work, and longer work hours [2]. A meta-analysis of research showed that female gender, not having children, and being single were all sociodemographic risk factors for experiencing burnout. Among the studies, burnout rates ranged from 15 to 90% showing a wide range of prevalence of burnout among health workers [2]. A systematic review of 100 studies in over 35 countries found that anxiety and depression among healthcare workers rose significantly post-pandemic [3]. The prevalence of anxiety among health care workers ranged from 22 to 36%, while depression rates ranged between 18 and 36% with the highest rates reported among physicians at a rate of 40% [3]. Additionally, studies have shown that healthcare workers continue to experience higher levels of anxiety and depression compared to before the pandemic [4]. These psychological effects increase the risk of burnout among health services occupations [3]. If burnout is not addressed, it can lead to anger and irritability, negative coping behaviors such as alcohol and substance abuse, and the development of chronic diseases [4]. Therefore, it is essential to recognize risk factors for burnout and employ strategies that both prevent the development of burnout and address burnout when it occurs [1].

Health worker burnout has been declared a current priority by the U.S. Surgeon General. One of the identified action steps for academic institutions to reduce health worker burnout is to “strengthen the connection between trainees and the communities they serve to mitigate burnout and build trust and connection” [5]. An academic college at a mid-sized university in the United States trains students in a myriad of health and service professions. The vision of the college is “to be an innovative leader in the student-centered, interprofessional preparation of health and human services professionals by providing students with an inclusive, community-engaged, and interdisciplinary academic experience” [6]. This study utilized components of *Photovoice*, a “process by which people can identify, represent and enhance their community through a specific photographic technique” to engage students with practicing professionals [7]. The study identified professionals’ perceptions of causes of burnout as well as factors that promote wellbeing by using photographs and quotes. 

The purpose of the study was to identify the skills/abilities needed to manage job-related stressors and prevent burnout and establish connections between students and the professional communities to give students a better understanding of the demands of their future professions.

## 2. Materials and Methods

Convenience sampling was used to identify community clinicians and practitioners in the areas of nursing, social work, speech language pathology, public health, healthcare administration, dietetics, physical therapy, athletic training, and dental hygiene. These fields were chosen based on their reported high rates of burnout and their inclusion in the U.S. Surgeon General’s identified priority audience of health workers. They were also chosen because training and academic programs in these fields are administered through the chosen academic college.

Prior to commencement, the study was approved by the university’s Institutional Review Board. Members of the research team from three applied research centers collaborated to identify and communicate with community practitioners. During initial communication, participants were informed of the study’s purpose as well as provided with guidelines for the appropriate use of photography. After providing written consent, community participants took part in a 10–15 min Zoom interview. Participants were asked, “What are the greatest sources of burnout in your career?” and “What factors promote your personal wellbeing and help you deal with difficult situations on the job?” Answers to these questions were recorded during the interview process through the Zoom Online Platform and the interview transcriptions were captured via closed captioning. The interview recordings were then uploaded to MAXQDA 2022 by VERBI software (2021) for thematic analysis. Following the interview, each community participant was asked to take a photograph that represented a source of burnout identified in the interview and a photograph that represented an identified factor promoting wellbeing. Following the submission of the photographs, the researcher and community participant identified a quote from the interview to accompany each photograph. 

After selection of the photos and quotes, they were enlarged and printed on 20 × 30 poster boards. They were displayed in a gallery-style exhibit for student viewing. The beginning of the exhibit included photographs and quotes representing factors related to burnout. Once students viewed the photos representing the negative aspects of their prospective careers, they then entered the gallery section showcasing factors that promote wellbeing. The participating community partners were standing beside their photos ready to discuss their experiences with students. This flow was intentional by presenting the potentially negative aspects of the career first, followed by sources of hope and opportunities for engagement. This format also allowed for connection and networking opportunities between students and community partners. At the conclusion of the study, participating community members were given $100 gift cards for their participation in the interviews, submission of photographs, and attendance at the student engagement event.

As students exited the event, they were asked to participate in a short survey administered through Qualtrics software, Version [November 2023] of Qualtrics. Copyright © [2020] Qualtrics. Qualtrics and all other Qualtrics product or service names are registered trademarks or trademarks of Qualtrics, Provo, UT, USA. https://www.qualtrics.com. Informed consent documents were presented prior to participating in the survey. A qualitative inquiry was used to gain a deeper understanding of the impact the Photovoice study had on students’ perceptions of the negative aspects of their prospective careers. Thematic analysis was chosen to analyze the data as it is flexible, accessible, and not aligned to one theoretical framework. A middle-ground approach was influenced by our knowledge of the existing literature, theories, and definitions from other contexts, as well as our experience of occupational burnout. Recognizing our subjectivity and our generational perspectives was important within the analysis, and valid interpretations were managed throughout by checking across three members of the research team. 

The first author familiarized herself with the data by reading responses on the single open-ended question. The data was then copied into a document format, annotating the data with reflections in the margins. Codes (units of meaning) were then generated line-by-line for all of the data. Following coding, codes were then grouped together under broader umbrella themes. The codes were checked for credibility by the second and third authors, who also identified some connections between the data and the relationship occupational burnout literature. This approach encompassed an inductive investigation of the data to identify repetitive themes, patterns, or concepts for the single open-ended question; “How did the event impact you?” Tags and labels were utilized to assign codes to the students’ answers to the single open-ended question. When the data were classified, we were able to identify the key words, patterns, and common themes in the textual data. Eligible students that chose to complete the survey were provided with a $20 gift card. Inclusion criteria included being 18 years of age or older and a registered student at the university.

## 3. Results

Thematic analysis was used to identify patterns across data derived from two open-ended items during interviews with community professionals and one open-ended question on how the Photovoice event impacted students. The process of coding took place in six phases: (1) became familiar with the data; (2) generated grid and initial codes; (3) searched for themes among the codes; (4) reviewed and defined themes; (5) named themes; and (6) reported final themes to other coders. Recognizing researcher subjectivity and researcher generational perspectives was important within the analysis, and valid interpretations were managed throughout by checking across members of the research team. 

### 3.1. Interviews with Community Professionals

Ten community professionals participated in the study. There were two professionals from nursing, and one each from physical therapy, public health, healthcare administration, social work, dietetics, athletic training, speech language pathology, and dental hygiene. The majority of the community professionals were female (80%) and Caucasian (90%). Three professionals were between the ages of 30 and 39 years old, four were between 40 49 years old, two were between 50 and 59 years old, and one was between 60 and 69 years old.

The themes that emerged from the data regarding factors that contribute to burnout in health services and professions were (1) Workload demands, (2) Unrealistic expectations, (3) Amount of time dedicated to care, and (4) Lack of work–life balance. See Table 1.

There were two most commonly identified factors contributing to burnout. The first was workload demands (n = 9). Lack of adequate staffing, administrative pressure to see large numbers of cases/patients, and increased number of required tasks in a workday were reported as contributors to burnout.

One participant quote echoed this theme: “Being overwhelmed with how many tasks there are to complete makes it very difficult to spend the time that you need to with patients”.

Another quote echoed this theme: “That’s definitely just the volume that gets pushed by a lot of employers in health care and I think that is due to what we see of just lower reimbursement rates to healthcare providers. I will say that is mostly driven by insurance companies”.

The other most commonly identified factor was unrealistic expectations (n = 9). Professionals expressed that unrealistic expectations from administration, patients, and self-imposed expectations all contribute to burnout.

One professional expressed this with the quote “I think perfectionism is a big driver for burnout because we strive to make sure all the i’s are dotted and t’s crossed. Then, when it doesn’t go the way you want, you are just pushing yourself to be perfect”.

Another quote also supported this theme along with several others: “It’s about the demands related to time and caseloads and client numbers and expectations. Also, it’s being present with people in some of the darkest or hardest moments of their lives”.

Another frequently mentioned factor contributing to burnout was the amount of time dedicated to care (n = 8). Participants indicated that the need to provide patient care even after the workday ends as well as being accessible all the time were drivers of burnout in health services professions.

One quote capturing this factor was “Ultimately, we stop when we are done with proper patient care and taking care of all the urgent and necessary requirements that the patient has or questions. So, not having a dedicated start and end time is primary reason for burnout across the board in healthcare because you can’t leave at the end of the day”.

Another quote supporting this theme: “I would probably say one of the biggest factors is now my normal day is I get here at 6:00 am and I am not going to leave until 7:30 tonight”.

Lack of work–life balance (n = 8) was also frequently mentioned in the interviews. The need to unplug and get away from work were listed as contributors to burnout, as well as the need to help everyone that asks. Several participants highlighted that often individuals that go into health services professions are “helpers”, so they consistently give of themselves even at their own detriment.

One quote captured this: “We’re in a position of service. So, I feel like every person we interact with, we give a little piece of ourselves to them. We are trying to help that person whether they understand that we are or not”.

Another quote also spoke to this theme: “We go into this because we like helping people. And there is such strong need for help, we become exhausted. But there’s always one more person that we want to help. Helpers aren’t always good at separating”.

The themes that emerged from the data regarding factors that promote well-being in health services professions were 1. Connecting with peers, 2. Physical activity, 3. Self- care, and 4. Identifying a mentor. See Table 2.

Again, there were two most commonly identified factors promoting well-being. The first factor was developing a strong peer group (n = 8). Most participants indicated the need to connect with peers in the same profession. This peer relationship allows for opportunities to empathize with one another and decompress without the need to explain all of the facets of the situation since both individuals have shared work experiences.

One professional said “I think it’s important to have somebody that you feel like is a really close friend that is in the same situation as you if at all possible, like a best friend at work. That relationship can offload so much of the stress by just being able to vent and get out some of the feelings that you’re having so that you’re just not internalizing them constantly”.

Another quote that supported this theme: “Talking with other people that are in the same boat as you and collaborating. I guess unloading your emotions. So, let me tell you about the day I had, especially if a patient dies or something, you really need an outlet to talk to somebody”.

The other most commonly identified factor was physical activity (n = 8). Outdoor activities, taking exercise classes, swimming, and lifting weights were all given as ways to promote well-being.

One participant captured the impact of physical activity by saying: “I started exercising and that has had a huge impact on my mental health. I was amazed after the first session. That I went in the car and turned the radio on and somebody said something funny on the radio and I laughed out loud and I was like, I haven’t heard myself laugh out loud in a long time. This exercise, there’s something to it. So, exercise is big for me”.

Another quote supporting this theme: “Also, I cannot overestimate the importance of exercise. As nurses, I feel like a lot of the time we don’t, we don’t focus on our own needs. Continuing to exercise and get out and do things that are good for you”.

Another frequently mentioned factor promoting well-being was self-care (n = 8). Methods of self-care listed were reading books, spending time with loved ones, pets, and meditating.

One professional expressed their thoughts on the importance of self-care on well-being by stating “We spend a lot of time teaching strategies to clients and vulnerable populations, but we don’t always employ those things with ourselves. And so, I think those are my most valuable things. One of those books I read once a year, just to remind myself of the strategies of how to protect myself, to shield myself, and to care for myself”.

Another quote supporting self-care: “I like to spend some time alone just reflecting on what it is that I like, like hobbies and pets and family. Just having something outside of work that helps you switch off and lets you come back after refreshing your mind”.

Identifying and connecting with a mentor (n = 5) was also frequently mentioned in the interviews. Having someone to assist with decision making, support you during difficult times, and share their past experiences with was identified as a protective factor with regard to burnout.

The role of a mentor was expressed by one participant by saying: “It’s really important to establish a mentor. In the field, someone that you trust and someone that you can feel good about talking to when you face situations where you may not feel confident in how you should move forward”.

Another quote regarding the importance of mentorship: “I had some great mentors in my late twenties who just really poured into me and helped me to start to understand what my purpose was and what my passion was. I just found fulfillment in that. I was able to lead in such a way that I truly understood my servant’s role”.

### 3.2. Results from Engagement Event

A total of 83 individuals completed the survey following the engagement event. Most participants identified as female (78%), White (75%), and non-Hispanic (86%). The majority of the participants were students (94%); other participants included community professionals (5%) and faculty (1%). Of the student participants, most were undergraduate students (88%) with the largest group comprised of college seniors (29%). Over half (59%) of the student participants were affiliated with programs related to health and human services. See Table 3. 

Again, thematic analysis was used to identify patterns across data derived from the one open-ended question on how the Photovoice event impacted participants. The themes that emerged from these data are 1. Learning self-care practices, 2. Gaining insight into the need for self-care, 3. A sense of connection, and 4. Exposure to different healthcare careers. See Table 4.

The most prominent theme students identified was learning different self-care practices when working in healthcare (n = 24). Specifically, students indicated they learned that stress reduction and maintaining work–life balance was critical to avoiding burnout in their prospective fields of work. Students identified specific techniques to utilize when experiencing stress and to avoid burnout. Among these techniques were investing time in hobbies and interests, exercising, listen to music, and prioritizing mental health.

One student quote exemplified this theme: “This event taught me how important it is to leave your work at work and focus on yourself to be healthy and happy”.

Another quote that emphasized this theme: “I learned how to separate work and life by using stress reduction techniques”.

The second most prominent theme was gaining insight into the need for self-care (n = 21). This theme was evident by students sharing their newly found insight that burnout is a serious health concern. The student comments that fell in this theme highlighted the realization that working in healthcare is overwhelming and the need for self-care is necessary to avoid inevitable burnout. For some students, the event caused them to look at their own stress and the need to take action.

One quote that emphasized this theme: ”This event helped me realize that I have to prioritize mental health and stress relief. After reading the posters, I now understand that I have to take part in self-care habits”.

Another quote that underscores this theme: “This event showed me the reality how working in healthcare is extremely draining and causes burnout but that there are ways to remedy this”.

The third theme that emerged from the student reflections on the impact of the Photovoice study was feeling connected (n = 17). Student perceptions indicated they felt a sense of connection when talking with professionals in the field of healthcare, especially if the professional was in their prospective field of study. Learning about their career of interest from actual professionals in the field was impactful. There was also another connection that students indicated, and this was feeling a sense of belonging. Students felt they were not alone in their own struggles with stress management. For these students, this connection gave them hope and confirmation that they are on the right path in their career pursuits.

One quote that emphasizes this theme: “Personally connecting with professionals and seeing that stress impacts them too impacted me the most”.

Another quote that underscores the theme of connection: “I got to talk to people in my career of interest that really knew what they were doing and loved it. It assured me that I am on the right path”.

The final theme that emerged from student reflections was learning about the different fields in healthcare (n = 15). Students shared their appreciation of being exposed to the various careers that are in healthcare which in turn, helped them consider more options than they previously had prior to the event. Student perceptions also indicated a newfound respect for the high levels of stress that is involved in healthcare careers. For some students, their impact also references the second theme regarding realization of the need for self-care.

One quote that emphasizes this theme: “I learned about different professions and how it effects them”.

Another quote that underscores this theme: “It allowed me to explore other areas of healthcare and learn more from experienced professionals”.

## 4. Discussion

Lacy et al. [8] found that the primary reasons given for burnout among 3000 healthcare workers were work-to-family conflicts, unrealistic expectations of patients, an ongoing pressure of continuous learning, long working hours, excessive bureaucracy, organizational issues, poor communication among healthcare professionals, and personal issues. The primary contributing factors of workload, unrealistic expectations, and lack of boundaries/work–life balance have been documented [9] and the results of the current study support these previous findings as workload demands, unrealistic expectations, time demands, and work–life balance were the most consistently reported factors contributing to burnout among this group of community practitioners. Therefore, results of this study were consistent with other research exploring factors contributing to burnout in health services professions. 

Additionally, the factors identified to promote wellbeing are also consistent with the literature regarding burnout prevention techniques among health workers as connection with peers, physical activity, self-care, and mentorship have been shown to be protective against burnout [10,11,12]. Studies [10,11] have supported the use of peer groups and development of mentorship among health service professions, which is consistent with the reported factors that contribute to well-being in this study. Physical activity has also been shown to have preventive effects on burnout [12]. The findings of this study support that physical activity is a commonly utilized method to reduce burnout among health services professionals. 

While the study met the goals of identifying the skills/abilities needed to manage job-related stressors and prevent burnout and establish connections between students and the professional communities, it was not without limitations. One limitation was selection bias of the community professionals that took part in the study. The community professionals were a convenience sample. Though the characteristics of the community participants were similar to the larger population of the health care workforce in our state, few males and few minorities chose to participate. Though the researchers oversampled minority community members for the study, there was a lack of participation from this group. This leads to the additional limitation of nonresponse bias. Another limitation was the sample size of community participants. There is potential that the factors related to burnout and coping identified by the group of participants do not represent the perceptions of a larger group of health care professionals. Though the research is qualitative, and the primary goal is not generalizability of the results, these factors are limitations when applying the findings to a broader context. 

University students’ involvement in experiential and community-based learning helps prepare them for life after graduation. These types of learning experiences allow for a better understanding of course content and deeper learning through application [13]. Students’ responses that the engagement event provided a sense of connection to community professionals, helped them feel seen, and fostered feelings of not being alone echoed the documented impacts of community-based learning. Through the engagement opportunity they also indicated that they felt more a part of their respective professional community. Research also has shown that students who perceive their individual skills and abilities in alignment with their future work are less likely to experience burnout and more likely to plan for sustained career engagement [14]. By connecting students with community health workers, students reported that they better understood the demands of their future professions. Additionally, they were more aware of resources and activities that promote wellbeing. 

To effectively manage burnout and promote wellbeing, there are individual- and organizational-level factors that must be addressed. Masalch [15] identified the following as ways to promote well-being at the workplace: “(a) a sustainable workload; (b) choice and control; (c) recognition and reward; (d) a supportive work community; (e) fairness, respect, and social justice; and (f) clear values and meaningful work.” As a university that trains students that will enter the health workforce, it is important to increase student awareness of these ways to prevent burnout so that they may consider them when job-seeking. The findings of this study also support the development of collegewide mentorship programs that could connect students approaching graduation with community professionals practicing in their respective career fields. A comprehensive approach is necessary to sustain a robust and healthy workforce.

## 5. Conclusions

This study found that health workers report that workload demands, unrealistic expectations, time, and lack of work/life balance are the main factors contributing to burnout in the health professions while connection with peers, physical activity, practicing selfcare, and identification of a mentor are factors that promote well-being. Additionally, it underscores findings that early exposure to self-care practices is necessary in the field of healthcare, as burnout seems inevitable. Student comments reflected the insight and realization that self-care practices are vital due to the toll healthcare careers have on mental health. The reality of burnout was conveyed and the need for work–life balance was received through the connections made between student and professional. 

Through community-engaged learning, this study was able to meet the objectives of identifying the skills/abilities needed to manage job-related stressors and prevent burnout and establish connections between students and the professional communities to give students a better understanding of the demands of their future professions. Furthermore, the study answered the U.S. Surgeon General’s call to action for academic institutions to strengthen the connections between student trainees and communities [5].

## Figures and Tables

**Table 1:** Main themes and subthemes regarding burnout identified from the interviews.

Main Theme	Subtheme	Sample Quote
Workload demands (n = 9) Workload demands focused on lack of adequate staffing, administrative pressure to see a large number of cases and/or patients, and increased number of required tasks in a workday.	Adequate Staffing	“Being overwhelmed with how many tasks there are to complete makes it very difficult to spend the time that you need to with patients”.
	Administrative Pressure	
	Increased Tasks	
Unrealistic expectations (n = 9) Unrealistic expectations speaks to unrealistic expectations from both administration and patients as well as self-imposed expectations.	Administrative Expectations	“I think perfectionism is a big driver for burnout because we strive to make sure all the i’s are dotted, and t’s crossed. Then, when it does not go the way you want, you are just pushing yourself to be perfect”.
	Patient Expectations	
	Perfectionism	
Amount of time dedicated to care (n = 8) Amount of time dedicated to care focused on the need to provide patient care even after the workday ends and being accessible to patients and/or clients all of the time.	Lack of “normal” working hours	“I would probably say one of the biggest factors is now my normal day is I get here at 6:00 am and I am not going to leave until 7:30 tonight”.
	24/7 Care	
Lack of life/work balance (n = 8) Lack of life/work balance speaks to the need to unplug and get away from work rather than giving from themselves at their own detriment.		“We go into this because we like helping people. And there is such a strong need for help, we become exhausted. But there’s always one more person that we want to help. Helpers are not always good at separating”.

**Table 2:** Main themes and subthemes regarding wellbeing identified from the interviews.

Main Theme	Subtheme	Sample Quote
Connecting with peers (n = 8) Connecting with peers focused on relationships with other professionals in the same field as well as being involved in professional organizations.	Relationships	“I think it’s important to have somebody that you feel like is a really close friend that is in the same situation as you, if at all possible, like a best friend at work. That relationship can offload so much of the stress by just being able to vent and get out some of the feelings that you’re having so that you’re just not internalizing them constantly”.
	Professional Organizations	
Physical activity (n = 8) Physical activity speaks to the importance of participating in outdoor activities as well as individual and group exercise activities and/or classes.	Outdoor Activities	“I started exercising and that has had a huge impact on my mental health. I was amazed after the first session. That I went in the car and turned the radio on, and somebody said something funny on the radio and I laughed out loud, and I was like, I have not heard myself laugh out loud in a long time. This exercise, there’s something to it. So, exercise is big for me”.
	Individual Exercise Activities/Classes	
	Group Exercise Activities/Classes	
Practicing self-care (n = 8) Practicing self-care focused on ways in which individuals participated in both solitary activities and reflection as well as group activities to decompress.	Solitary Activities	“I like to spend some time alone just reflecting on what it is that I like, like hobbies and pets and family. Just having something outside of work that helps you switch off and lets you come back after refreshing your mind”.
	Loved Ones	
	Faith-based Practices	
Identification of a mentor (n = 5) Identification of a mentor speaks to the importance of connecting with an administrator for support as well as senior employees in order to help with decision making.	Administrator Support	“It’s really important to establish a mentor. In the field, someone that you trust and someone that you can feel good about talking to when you face situations where you may not feel confident in how you should move forward”.
	Senior Employee	

**Table 3:** Engagement event participant characteristics.

Characteristic	n (%)
Gender	
Female	65 (78.3)
Male	17 (20.5)
Non-binary	1 (1.2)
Race	
White	62 (74.7)
Black or African American	10 (12.1)
Asian	6 (7.2)
Prefer not to answer	5 (6.0)
Ethnicity	
Non-Hispanic	71 (85.5)
Hispanic	11 (13.3)
Prefer not to answer	1 (1.2)
Student Classification	
Undergraduate	58 (86.8)
Graduate	9 (13.2)

**Table 4:** Main themes and subthemes regarding the impact of the engagement event.

Main Theme	Subtheme
Practicing self-care	Establishing work–life balance
	Investing time in hobbies and interests
	Listening to music
Gaining insight into the need for self-care	Exercising
	Prioritizing mental health
	Realization that working in healthcare is overwhelming
	Need for self-care is necessary to avoid
	inevitable burnout Examination of one’s own stress and the need to take action
A sense of connection	Talking with professionals in the health field Connecting with professionals in their future careers
	Sense of belonging
	Feelings of not being alone, shared stressors
Exposure to different healthcare careers	Increased knowledge of different health services fields
	Consideration of additional career options Newfound respect for health services professionals

## Data Availability

Due to the ability to identify participants, data are available upon request.
